# The Impact of COVID-19 Infection on the Development of Stroke, Pulmonary Embolism, and Myocardial Infarction: A Retrospective Study

**DOI:** 10.7759/cureus.77665

**Published:** 2025-01-19

**Authors:** Oya Güven, Gökhan Karakurt, Abdulrahman Naser, Hakan Selçuk, Dilek V Keleş, Emre Gedik, Mert Avsever, Fatih Furkan Köse

**Affiliations:** 1 Department of Emergency Medicine, Kırklareli University Faculty of Medicine, Kırklareli, TUR; 2 Department of Emergency Medicine, Kırklareli Training and Research Hospital, Kırklareli, TUR; 3 Department of Pulmonology, Kırklareli Training and Research Hospital, Kırklareli, TUR; 4 Department of Cardiology, Kırklareli Training and Research Hospital, Kırklareli, TUR; 5 Department of Emergency Medicine, Babaeskı State Hospital, Kırklareli, TUR; 6 Department of Nursing, Kırklareli University Faculty of Health Sciences, Kırklareli, TUR; 7 Department of Neurology, Kırklareli Training and Research Hospital, Kırklareli, TUR; 8 Department of Emergency Medicine, St. John's Hospital, Edinburgh, GBR

**Keywords:** covid-19, myocardial infarction, pulmonary embolism, stroke, vaccine

## Abstract

Introduction: This study compares the period during which thromboembolic disease develops after contact with the virus before, during, and after the pandemic.

Methods: In this study, the medical records of patients with a preliminary diagnosis of myocardial infarction (MI), pulmonary embolism (PE), and ischemic stroke who presented to the Emergency Department before, during, and after the pandemic (when vaccination rates increased) were retrospectively examined. Data on whether these patients had COVID-19 or were vaccinated, the time interval between infection/vaccination and the onset of these conditions, and the prognosis were analyzed.

Results: In the MI group, patients developed embolism the longest after infection and the shortest after vaccination. Among MI patients, the rate of those who received the BioNTech vaccine during the normalization period was higher than that of those who received Sinovac (p = 0.005). In stroke patients, during the pandemic, the time to post-vaccine embolism was shorter (p < 0.001). Additionally, infection and vaccination increased the mortality rate in stroke and PE patients (p < 0.001).

Conclusion: This study demonstrates that thromboembolic events can occur at varying rates and durations after exposure to the virus. While the causes of thrombosis are multifactorial, contact with the virus may act as a triggering factor, even if COVID-19 does not have a direct effect.

## Introduction

The severe acute respiratory syndrome coronavirus 2 (SARS-CoV-2) virus, a member of the Coronaviridae family, is characterized as a positive-sense, single-stranded RNA virus. It emerged as the causative agent of COVID-19, triggering a global pandemic toward the end of 2019 [1]. Initially, it was primarily implicated in mortality related to respiratory failure during respiratory tract infections. Subsequent research has elucidated its pivotal role in hyperinflammation, endothelial damage, and activation of the coagulation cascade [2, 3]. Although extensive research has been conducted on the pathogenesis of SARS-CoV-2, certain aspects remain unclear. Notably, in numerous cases of severe pneumonia attributable to the virus, an increase in coagulation activity and a concomitant rise in ischemic conditions have been observed [4].

During infection, elevated inflammation parameters and cytokine levels, coupled with endothelial damage caused by the virus's direct infection of endothelial cells, lead to intravascular damage and set the stage for hypercoagulation [5]. This is compounded by the development of hyperviscosity, initiating a vicious cycle that culminates in thrombotic events [6]. SARS-CoV-2 possesses spike proteins on its surface, which interact with specific receptors on the surfaces of target host cells, entering via the angiotensin-converting enzyme 2 (ACE2) receptor [7, 8]. This receptor is highly expressed in heart cells, vascular endothelium, type 2 alveolar cells, and ciliary and goblet cells in the respiratory tract [9], indicating that these organs are primarily affected. Furthermore, studies suggest that inflammation-induced vasculitis in the vascular endothelium, conditions such as myocarditis, and an increased risk of arrhythmia and myocardial infarction (MI) are associated with COVID-19 [10]. Particularly in elderly populations with pre-existing chronic diseases and already damaged vessels, the risk of thrombosis may be heightened by COVID-19 infection. Tissue hypoxia further contributes to this, increasing the risk of cardioembolic stroke [11]. The high mortality rate in patients with coagulopathy highlights the significant role of coagulation in the pathogenesis of COVID-19. Hypercoagulation can lead to severe complications such as MI, pulmonary embolism (PE), stroke, arterial and venous thrombosis, and miscarriage [12]. Notably, administering anticoagulant therapy to patients with D-dimer levels above 3 μg/mL has shown positive outcomes, reducing mortality and confirming these observations [13].

Numerous studies in the medical literature have demonstrated the thrombotic effects of COVID-19 [14-16]. This study aims to investigate the impact of SARS-CoV-2 on the development of thromboembolic disease following exposure to the virus. It includes a comparative analysis of the timeframes for the development of thromboembolic disease before, during, and after the pandemic (as the impact of the pandemic has waned).

## Materials and methods

Study design

This study retrospectively examined the records of patients who presented to the Emergency Department (ED) between January 2019 and December 2021. These patients were initially diagnosed with MI, PE, or ischemic stroke, and their final diagnoses were confirmed through consultations with the departments of pulmonology, cardiology, radiology, and neurology. The study focused on whether these patients had contracted COVID-19 or received a COVID-19 vaccine, their polymerase chain reaction (PCR) test results, the time elapsed since infection/vaccination before developing these conditions, and their prognoses. None of the patients were using anticoagulants or antiplatelet therapy.

Time frame

Patient admissions were classified based on the first COVID-19 case reported in our country, starting from March 2020. The period before this date was designated as the pre-pandemic period (January 1, 2019, to February 29, 2020, 14 months). The pandemic period extended from March 1, 2020, to June 30, 2021 (16 months), during which the second dose of vaccination was completed. The normalization period was from July 1, 2021, to December 31, 2021 (6 months), when quarantine measures were eased. In our country, Sinovac and BioNTech vaccines were administered, and the study's vaccination rates were based on these vaccine types.

Diagnostic criteria

According to the European Cardiology Association guidelines [17], the diagnosis of MI was confirmed by ST elevation on electrocardiography (ECG), representing the acute coronary syndrome subtype associated with the greatest inflammatory response and thrombus accumulation, which were included in this study. Ischemic stroke was diagnosed by diffusion restriction on diffusion-weighted magnetic resonance imaging (DWI-MRI), and PE was diagnosed by filling defects on computed tomography pulmonary angiogram (CTPA). A radiologist evaluated and reported the images, which were subsequently reviewed and approved by neurology and pulmonology specialists.

Patients with chest pain and elevated troponin but no ST elevation on ECG, brain CT showing hemorrhage, and elevated D-dimer but no PE on CTPA were excluded from the study.

Statistical methods

The descriptive statistics of the data included mean, standard deviation, minimum, maximum, frequency, and ratio values. Categorical data were expressed as numbers and percentages, while continuous data with a normal distribution were expressed as mean ± standard deviation, and continuous data without a normal distribution were expressed as median (minimum-maximum). The Kolmogorov-Smirnov test was used to evaluate the distribution of variables. Quantitative independent data were analyzed using ANOVA (Tukey test), independent sample t-test, and Mann-Whitney U-test. The chi-square test was used to analyze qualitative independent data. A p-value <0.05 was considered statistically significant in all tests. Statistical analyses were performed using IBM SPSS Statistics for Windows, Version 28 (Released 2021; IBM Corp., Armonk, New York).

Ethical approval

This study received ethical approval from the Kırklareli University Faculty of Medicine Scientific Research Ethics Committee (P202200014/01-26.05.22). Due to its retrospective nature, declarations of human ethics and consent to participate were not applicable.

## Results

The study identified 263 MI, 785 stroke, and 99 PE patients who met the criteria. Of these, 469 (40.8%) were female. A total of 704 (61.3%) patients presented during the pandemic and normalization periods, with 33 (4.6%) testing positive on PCR tests. Patients in the MI group developed embolism the longest after COVID-19 infection (median: 275 days) and the shortest after vaccination (median: 110 days). A significant proportion of stroke patients (31.6%) were discharged; however, the median duration from admission to mortality was notably brief, at four days (Table 1).

**Table 1:** General characteristics of the patients *It was identified that patients had multiple comorbidities and received various types of vaccinations. MI: myocardial infarction; PE: pulmonary embolism; COPD: chronic obstructive pulmonary disease; DM: diabetes mellitus; CKD: chronic kidney disease; PCR: polymerase chain reaction; ED: emergency department.

Variables	MI	Stroke	PE
Age, mean±SD (years)	61.2±13.4	73.1±13.2	65.7±18.2
Gender, n (%)			
Female	62 (23.6%)	353 (45.0%)	54 (54.5%)
Male	201 (76.4%)	432 (55.0%)	45 (45.5%)
Comorbidity*, n (%)			
Hypertension	141 (53.6%)	573 (73.0%)	40 (40.4%)
Arrhythmia	13 (4.9%)	180 (22.9%)	9 (9.1%)
Heart failure	71 (27.0%)	103 (13.1%)	16 (16.2%)
Hyperlipidemia	100 (38%)	121 (15.4%)	4 (4.0%)
COPD	26 (9.9%)	65 (8.3%)	11 (11.1%)
DM	73 (27.8%)	205 (26.1%)	16 (16.2%)
Cancer	5 (1.9%)	38 (4.8%)	10 (10.1%)
CKD	5 (1.9%)	36 (4.6%)	-
Chronic liver disease	1 (0.4%)	1 (0.1%)	-
PCR, n (%)			
Negative	142 (94.0%)	484 (96.8%)	45 (84.9%)
Positive	9 (6.0%)	16 (3.2%)	8 (15.1%)
Thromboembolism duration, mean±SD	220.4±133.7	115.8±128.2	65.6±85.0
Vaccine, n (%)			
Negative	96 (63.6%)	291 (58.2%)	31 (58.5%)
Positive	55 (36.4%)	209 (41.8%)	22 (41.5%)
Vaccine type*, n (%)			
Sinovac	32-47.7%	190-78.1%	17-65.3%
BioNTech	35-52.3%	53-21.9%	9-34.7%
Post-vaccination period, mean±SD	116.0±67.3	119.5±76.3	145.8±94.6
Outcomes in the ED, n (%)			
Discharge	-	248-31.6%	18-18.2%
Intensive care unit	238-90.5%	110-14.0%	33-33.3%
Service	-	353-45.0%	43-43.4%
Referred	25-9.5%	74-9.4%	5-5.1%
Outcome, n (%)			
Discharged	232-97.5%	321-60.8%	48-63.2%
Expired	6-2.5%	143-39.2%	28-36.8%
Number of days until death, mean±SD	12.0±18.2	7.9±11.9	9.1±13.2

**Table 2:** Comparison of patient data according to periods *It was identified that patients received various types of vaccinations. ^a^Difference with the pre-pandemic period significant at p<0.05. ^b^Difference with the pandemic period significant at p<0.05. ^c^ANOVA. ^d^Independent sample t-test. ^e^Mann-Whitney U-test. ^f^Chi-square test (Fisher's test). ^g^Kruskal-Wallis test. MI: myocardial infarction, PCR: polymerase chain reaction, ED: emergency department, SD: standard deviation.

Variables	Pre-pandemic	Pandemic	Post-pandemic	p
MI				
Age, mean±SD (years)	61.9±13.4	59.8±13.5	62.7±13.3	0.353^c^
Gender, n (%)				
Female	27 (24.1%)	25 (24.3%)	10 (20.8%)	0.884^f^
Male	85 (75.9%)	78 (75.7%)	38 (79.2%)	
PCR, n (%)				
Negative	-	100 (97.1%)	42 (87.5%)	0.020^f^
Positive	-	3 (2.9%)	6^b^ (12.5%)	
Number of days elapsed post-infection, mean±SD	-	143.3±212.8	259.0±71.7	0.245^d^
Vaccine, n (%)				
Negative	-	92 (89.3%)	4 (8.3%)	<0.005^f^
Positive	-	11 (10.7%)	44 (91.7%)	
Vaccine type*, n (%)				
Sinovac	-	8 (72.7%)	24 (42.8%)	0.274^f^
BioNTech	-	3 (27.3%)	32 (57.2%)	0.005^f^
Number of days elapsed post-vaccine, mean±SD	-	47.6±44.4	133.1±61.2	<0.005^d^
Outcomes in the ED, n (%)				
Discharge	-	-	-	0.646^f^
Intensive care unit	101 (90.2%)	95 (92.2%)	42 (87.5%)	
Service	-	-	-	
Referred	11 (9.8%)	8 (7.8%)	6 (12.5%)	
Outcome, n (%)				
Discharged	99 (98.0%)	91 (95.8%)	42 (100%)	>0.05^f^
Expired	2 (2.0%)	4 (4.2%)	0 (0%)	
Number of days until death, mean±SD	1.0±0.0	17.5±20.8		0.140^e^
Stroke				
Age, mean±SD (years)	73.3±13.0	72.6±13.4	73.8±13.0	0.507^g^
Gender, n (%)				
Female	145 (50.9%)	152 (41.1%)	56 (43.1%)	0.039^f^
Male	140 (49.1%)	218 (58.9%)	74 (56.9%)	
PCR, n (%)				
Negative	-	360 (97.3%)	124 (95.4%)	0.286^f^
Positive	-	10 (2.7%)	6 (4.6%)	
Number of days elapsed post-infection, mean±SD		84.2±95.5	168.3±166.1	0.378^e^
Vaccine, n (%)				
Negative	-	279 (75.4%)	12 (9.2%)	<0.005^f^
Positive	-	91 (24.6%)	118 (90.8%)	
Vaccine type*, n (%)				
Sinovac	-	90 (98.9%)	100 (65.7%)	<0.005^f^
BioNTech	-	1 (1.1%)	52 (44.3%)	<0.005^f^
Number of days elapsed post-vaccine, mean±SD		74.8±41.3	153.6±79.3	<0.005^d^
Outcomes in the ED, n (%)				
Discharge	73 (25.6%)	135 (36.5%)	40 (30.8%)	<0.005^f^
Intensive care unit	40 (14.0%)	55 (14.9%)	15 (11.5%)	
Service	154 (54.0%)	145 (39.2%)	54 (41.5%)	
Referred	18 (6.3%)	35 (9.5%)	21 (16.2%)	
Outcome, n (%)				
Discharged	174 (89.7%)	139 (69.5%0	7 (10.1%)	<0.005^f^
Expired	20 (10.3%)	61^a^ (30.5%)	62^a,b^ (89.9%)	
Number of days until death, mean±SD	17.5±21.8	6.7±10.7	6.3±6.5	0.026^g^
PE				
Age, mean±SD (years)	66.4±17.6	62.9±18.3	68.9±19.5	0.375^g^
Gender, n (%)				
Female	25 (54.3%)	17 (51.5%)	12 (60.0%)	0.834^f^
Male	21 (45.7%)	16 (48.5%)	8 (40.0%)	
PCR, n (%)				
Negative	-	29 (87.9%)	16 (80.0%)	0.437^f^
Positive	-	4 (12.1%)	4 (20.0%)	
Number of days elapsed post-infection, mean±SD		19.5±18.9	111.8±104.1	0.110^e^
Vaccine, n (%)				
Negative	-	29 (87.9%)	2 (10.0%)	<0.005^f^
Positive	-	4 (12.1%)	18 (90.0%)	
Vaccine type*, n (%)				
Sinovac	-	4 (100%)	13 (59.0%)	0.535^f^
BioNTech	-	0 (0)	9 (41.0%)	0.115^f^
Number of days elapsed post-vaccine, mean±SD		112.3±70.0	153.2±99.3	0.447^d^
Outcomes in the ED, n (%)				
Discharge	10 (21.7%)	5 (15.2%)	3 (15%)	0.388^f^
Intensive care unit	13 (28.3%)	11 (33.3%)	9 (45%)	
Service	20 (43.5%)	17 (51.5%)	6 (30%)	
Referred	3 (6.5%)	0 (0%)	2 (10%)	
Outcome, n (%)				
Discharged	30 (90.9%)	17 (60.7%)	1 (6.7%)	<0.005^f^
Expired	3 (9.1%)	11^a^ (39.3%)	14^a,b^ (93.3%)	
Number of days until death, mean±SD	35.3±25.4	3.5±2.4	7.8±8.4	0.020^g^

When comparing patients across different periods, it was observed that for MI patients, the number of cases during the normalization period was significantly lower (18.2%) compared to other periods. During this period, the proportion of patients who had contracted COVID-19 (p = 0.020) and those who had been vaccinated (p < 0.001) was higher than in other periods. Additionally, during the normalization period, the rate of individuals vaccinated with the BioNTech vaccine was higher than with the Sinovac vaccine (p = 0.005). The time to embolism post-vaccination during the pandemic period was shorter than in other periods (median: 37.0 days). However, the time between contracting COVID-19 and the occurrence of embolism was not significant (p = 0.245) (Table 2).

For stroke patients, the number of cases during the pandemic period (47.1%) and the rate of thromboembolism in males (p = 0.039) were higher compared to other periods. The proportion of vaccinated individuals during the normalization period, particularly those vaccinated with the BioNTech vaccine, was significantly higher than in the previous period (p < 0.001). Following vaccination during the pandemic, the time to embolism was shorter (p < 0.001). No relationship was detected between contracting COVID-19 and the occurrence of embolism (p = 0.378). The proportion of patients who expired during the normalization period was higher than in other periods (p < 0.001). Additionally, the time to death following disease onset during the pandemic was shorter than in other periods (p = 0.026) (Table 2).

For PE patients, the vaccination rate during the normalization period was higher than in other periods (p < 0.001). There was no significant difference between the type of vaccine received and the time to embolism following vaccination (p > 0.005). The mortality rate during the normalization period was higher than in other periods (p < 0.001). Following embolism during the pandemic, the time to death was shorter than in other periods (p = 0.020) (Table 2).

In stroke patients, the average age of the deceased group was significantly higher compared to other groups (p = 0.001). In the MI group, a large portion of deceased patients were male, whereas in the stroke and PE groups, the number of deceased female patients was higher. Across all patient cohorts with fatalities, a significantly elevated prevalence of comorbid conditions was observed. For stroke and PE patients, it was determined that having COVID-19 and being vaccinated increased the mortality rate (p < 0.001). There was no significant difference in the time elapsed between contracting COVID-19 and death among the three groups (Table 3).

**Table 3:** Comparison of data according to the prognosis of patients ^a^Independent sample t-test. ^b^Chi-square test (Fisher's test). ^c^Mann-Whitney U-test. Ex (+): exitus group, Ex (-): survivor group, MI: myocardial infarction, PE: pulmonary embolism, COVID: coronavirus disease, SD: standard deviation.

Variables	Ex (-)	Ex (+)	p
MI			
Age, mean±SD	61.1±13.5	65.5±8.0	0.430^a^
Gender, n (%)			
Female	60 (23.3%)	2 (33.3%)	0.628^b^
Male	197 (76.7%)	4 (66.7%)	
Comorbidity, n (%)			
Negative	94 (36.6%)	2 (33.3%)	0.870^b^
Positive	163 (63.4%)	4 (66.7%)	
COVID, n (%)			
Negative	249 (96.9%)	5 (83.3%)	0.190^b^
Positive	8 (3.1%)	1 (16.7%)	
Number of days elapsed post-infection, mean±SD	247.9±112.7	1.0±1.0	0.078^a^
Vaccine, n (%)			
Negative	202 (78.6%)	6 (100%)	0.349^b^
Positive	55 (21.4%)	0 (0%)	
Number of days elapsed post-vaccine, mean±SD	116.0±67.3	-	-
Stroke			
Age, mean±SD	72.3±13.4	76.4±11.8	0.001^c^
Gender, n (%)			
Female	277 (43.2%)	76 (52.8%)	0.037^b^
Male	364 (56.8%)	68 (47.2%)	
Comorbidity, n (%)			
Negative	76 (11.9%)	21 (14.6%)	0.369^b^
Positive	565 (88.1%)	123 (85.4%)	
COVID, n (%)			
Negative	635 (99.1%)	134 (93.1%)	<0.005^b^
Positive	6 (0.9%)	10 (6.9%)	
Number of days elapsed post-infection, mean±SD	133.8±147.1	104.9±122.6	0.826^c^
Vaccine, n (%)			
Negative	540 (84.2%)	36 (25%)	<0.005^b^
Positive	101 (15.8%)	108 (75%)	
Number of days elapsed post-vaccine, mean±SD	115.2±74.4	123.5±78.0	0.425^c^
PE			
Age, mean±SD	63.7±18.0	70.8±18.1	0.054^c^
Gender, n (%)			
Female	36 (50.7%)	18 (64.3%)	0.222^b^
Male	35 (49.3%)	10 (35.7%)	
Comorbidity, n (%)			
Negative	27 (38.0%)	8 (28.6%)	0.375^b^
Positive	44 (62.0%)	20 (71.4%)	
COVID, n (%)			
Negative	70 (98.6%)	21 (75%)	<0.005^b^
Positive	1 (1.4%)	7 (25%)	
Number of days elapsed post-infection, mean±SD	28.0±28.0	71.0±90.4	0.661^c^
Vaccine, n (%)			
Negative	66 (93.0%)	11 (39.3%)	<0.005^b^
Positive	5 (7.0%)	17 (60.7%)	
Number of days elapsed post-vaccine, mean±SD	197.0±111.8	130.7±86.9	0.174^a^

In stroke patients, those who received the Sinovac vaccine demonstrated higher protection against COVID-19 infection compared to those who received the BioNTech vaccine. Both types of vaccination offered similar levels of protection in patients with MI and PE. However, in stroke patients, the elapsed time to stroke was longer for those who received the BioNTech vaccine than for those who received the Sinovac vaccine. Conversely, in PE patients, those who received the Sinovac vaccine had a longer elapsed time to PE compared to those who received the BioNTech vaccine (Table 4).

**Table 4:** Prognosis according to vaccine types The group that received both vaccines was not analyzed. ^a^Mann-Whitney U-test. ^b^Chi-square test (Fisher's test). ^c^Independent sample t-test. MI: myocardial infarction, COVID: coronavirus disease, PE: pulmonary embolism, SD: standard deviation.

Variables	Sinovac	BioNTech	p
MI			
Age, mean±SD	65.4±13.2	56.3±12.6	0.027^a^
Gender, n (%)			
Female	10 (50.0)	3 (13.0)	0.008^b^
Male	10 (50.0)	20 (87.0)	
COVID, n (%)			
Positive	2 (10.0)	5 (21.7)	0.298^b^
Negative	18 (90.0)	18 (78.3)	
Number of days elapsed post-infection, mean±SD	261.0±179.6	284.0±41.6	0.886^a^
Number of days elapsed post-vaccine, mean±SD	129.5±84.1	113.8±62.9	0.488^a^
Stroke			
Age, mean±SD	76.9±10.1	55.5±13.0	<0.005^a^
Gender, n (%)			
Female	79 (50.6)	5 (26.3)	0.045^b^
Male	77 (49.4)	14 (73.7)	
COVID, n (%)			
Positive	5 (3.2)	3 (15.8)	0.043^b^
Negative	151 (96.8)	16 (84.2)	
Number of days elapsed post-infection, mean±SD	12.4±22.8	318.3±24.7	0.022^a^
Number of days elapsed post-vaccine, mean±SD	129.0±79.7	101.8±67.1	0.230^a^
PE			
Age, mean±SD	80.1±10.4	46.0±10.3	<0.005^c^
Gender, n (%)			
Female	9 (69.2)	2 (40.0)	0.326^b^
Male	4 (30.8)	3 (60.0)	
COVID, n (%)			
Positive	2 (15.4)	1 (20.0)	1.000^b^
Negative	11 (84.6)	4 (80.0)	
Number of days elapsed post-infection, mean±SD	14.5±19.1	250.0±0.0	0.063^c^
Number of days elapsed post-vaccine, mean±SD	201.1±81.6	66.2±42.0	0.003^c^

## Discussion

This study aimed to compare the timeline of disease development with high and low viral loads, where thrombosis plays a significant role in the pathophysiology. The findings suggest that viral states can trigger such diseases. The study also demonstrated a correlation between the virus and mortality from thromboembolic events. Furthermore, it found that vaccination can provide protection against these diseases.

In a study conducted by Taylor et al., it was reported that 27% of patients who experienced a stroke in conjunction with COVID-19 infection were under the age of 50 years [18]. However, due to the small sample size in their study, generalizing these findings is challenging. In contrast, our study identified that while the average age of all patients with thromboembolic disease was over 60 years, embolisms could develop even in patients as young as 19 years. Despite a higher average age compared to other studies, the undeniable reality is that COVID-19 can cause pathology even in young individuals without chronic diseases.

Some studies have found that women are more likely to contract COVID-19 than men but have a lower mortality rate. It is believed that the additional X chromosome in women contributes to immune diversity, creating a protective mechanism [19]. Other studies have determined that men are more susceptible to COVID-19 infection [20]. While some studies suggest the risk of developing thromboembolism is the same in both sexes, others indicate that women are at a higher risk [21-23]. In this study, the rate of COVID-19 infection was lower in women. The risk of developing thromboembolism, excluding PE, was lower in women, but the mortality rate, excluding MI, was higher. Based on these findings, it can be concluded that the risk of infection or developing embolism based on gender remains unclear.

Studies have shown that hospital admissions due to MI decreased during the pandemic [24]. This has been attributed to strict quarantine measures and the fear among individuals with chronic diseases of contracting infections in hospitals. However, this study found no significant decrease in patients with MI, stroke, and PE during the pandemic compared to the pre-pandemic period. On the contrary, a notable decrease in cases was observed during the normalization period. Despite an increase in the regional population and emergency service admissions during the normalization period, the development of thrombotic diseases was reduced. This suggests that during the pandemic, the vulnerable population may have experienced fatalities outside hospitals due to the virus's impact [25]. The low number of PCR-positive cases indicates that there may not be a direct relationship between COVID-19 and the development of thromboembolic diseases. However, the mortality rate increased during the pandemic compared to the pre-pandemic period. In PE patients, thrombotic events occurred shortly after COVID-19 infection, suggesting a high affinity of the virus for the pulmonary artery, possibly mediated by the ACE2 receptor. The duration of PE following vaccination was notably longer than in the other groups. This finding highlights the vaccine's effectiveness, particularly with Sinovac, which demonstrates clear protective benefits in PE patients. The mortality rate in the infection and vaccination groups was higher in the PE and stroke groups. The time to embolism occurrence in MI and stroke patients was identified as 30-60 days. The shortest time to thrombotic event occurrence post-vaccination was observed in MI patients. The vaccination rate was the lowest in MI patients compared to the other groups. Although a higher mortality rate was observed in the non-vaccinated group of MI patients, no significant difference was found compared to the vaccinated group. Furthermore, during the normalization phase, characterized by elevated vaccination rates, a notable prolongation in the time to embolic event manifestation was observed among MI patients. In stroke patients, the proportion of those who experienced an infection and were vaccinated was significantly higher in the deceased group. According to this research, the probability of mortality among embolism patients is significantly higher in cases of infections. Conversely, vaccination has been shown to reduce the mortality rate in MI cases. Additionally, this study observed a notable decrease in the duration from disease onset to mortality during the pandemic, except in MI cases. This implies that the severity of seasonal viral load may impact the mortality rate. 

Since the pandemic started, the elderly have been prioritized for Sinovac vaccination in our country. This focus on the elderly likely accounts for the higher average age of individuals vaccinated with Sinovac across all demographic groups.

In one study, no thromboembolic events were observed within 14 days in individuals vaccinated with BioNTech [26]. This study also found that heightened uptake of BioNTech vaccination was associated with a delayed onset of MI and stroke events during the normalization period. The consistent vaccination rates with Sinovac and BioNTech among PE patients across normalization periods, without influencing the temporal progression to thrombosis, support this hypothesis. Accordingly, BioNTech vaccination provides protection against both infection and COVID-19's thrombotic effects. Additionally, in this study, the time to develop thromboembolism after COVID-19 infection or vaccination was over 30 days. Another study found that patients who had recovered from COVID-19 continued to show increased thrombin formation in blood tests even four months later [27], suggesting the impact of long COVID. This implies that anticoagulant therapy could be considered for both acute and chronic phases, not only for those who have had an infection but also for those vaccinated.

A study found that patients with comorbidities, particularly those with hypertension, diabetes, and arrhythmias, exhibited a higher mortality rate when infected with COVID-19 [28]. Similarly, this study identified that patients with hypertension had the highest incidence of thromboembolism and experienced greater mortality compared to patients with other conditions. The presence of multiple comorbidities in the elderly population, along with a history of polypharmacy, care needs, and diminished immunity, may increase susceptibility to COVID-19. It has been observed that COVID-19 has a prothrombotic nature, which increases the risk of blood clotting and embolism. This biological mechanism contributes to the increased mortality associated with the virus. Additionally, recent findings suggest that the interval from infection to mortality is reduced during periods of elevated viral load, further supporting this hypothesis.

The incidence of thromboembolic events in COVID-19 patients is higher than in other influenza-like illnesses. However, it is crucial to note that the occurrence of these conditions is multifactorial, and it would be incorrect to establish direct causation with the virus [29]. Thromboembolic events following exposure to the virus clearly indicate that COVID-19 acts as a triggering factor rather than directly causing these events.

Limitations

The primary limitation of this study is its retrospective nature and single-center design. Blood tests for hypercoagulability (such as D-dimer levels) were not examined; instead, the focus was on diagnostic procedures (such as CT scans and ECGs). This approach allowed for data analysis from patients with confirmed diagnoses, yielding statistical results on the likelihood of thrombosis development following COVID-19 infection. Although the number of patients with a positive PCR test was small, the undeniable impact of viral load during the pandemic on patients and disease types cannot be overlooked. The prognosis of patients referred to higher-level centers could not be determined. Additionally, predispositions to hypercoagulability, such as a sedentary lifestyle, obesity, smoking, substance use history, deep vein thrombosis, pregnancy, or history of immobility, and the presence of undiagnosed conditions, were not accounted for. The size of the thrombus and the extent of vascular occlusion were also not determined. Because the study did not record the type of treatment received for chronic conditions (such as ACE inhibitors), it was not possible to further differentiate the affinity of SARS-CoV-2. It remains uncertain if other factors could act as prognostic indicators. However, the study's findings remain applicable, as it includes data from the largest and sole hospital in the area.

## Conclusions

Patients with a history of MI, stroke, or PE who contract a COVID-19 infection or receive a vaccination must be closely monitored for signs and symptoms of embolic events. Further research is needed to establish clearer connections between COVID-19 and thromboembolic events. Such studies are essential to mitigate risks effectively by strategically timing vaccinations for at-risk patients, selecting appropriate vaccine types, and implementing rigorous monitoring protocols following vaccination. These research efforts will provide valuable insights into the pandemic's far-reaching implications and enable evidence-based decision-making.
